# Killing Them with Kindness? In-Hive Medications May Inhibit Xenobiotic Efflux Transporters and Endanger Honey Bees

**DOI:** 10.1371/journal.pone.0026796

**Published:** 2011-11-02

**Authors:** David J. Hawthorne, Galen P. Dively

**Affiliations:** Department of Entomology, University of Maryland, College Park, Maryland, United States of America; Ghent University, Belgium

## Abstract

**Background:**

Honey bees (*Apis mellifera*) have recently experienced higher than normal overwintering colony losses. Many factors have been evoked to explain the losses, among which are the presence of residues of pesticides and veterinary products in hives. Multiple residues are present at the same time, though most often in low concentrations so that no single product has yet been associated with losses. Involvement of a combination of residues to losses may however not be excluded. To understand the impact of an exposure to combined residues on honey bees, we propose a mechanism-based strategy, focusing here on Multi-Drug Resistance (MDR) transporters as mediators of those interactions.

**Methodology/Principal Findings:**

Using whole-animal bioassays, we demonstrate through inhibition by verapamil that the widely used organophosphate and pyrethroid acaricides coumaphos and τ-fluvalinate, and three neonicotinoid insecticides: imidacloprid, acetamiprid and thiacloprid are substrates of one or more MDR transporters. Among the candidate inhibitors of honey bee MDR transporters is the in-hive antibiotic oxytetracycline. Bees prefed oxytetracycline were significantly sensitized to the acaricides coumaphos and τ-fluvalinate, suggesting that the antibiotic may interfere with the normal excretion or metabolism of these pesticides.

**Conclusions/Significance:**

Many bee hives receive regular treatments of oxytetracycline and acaricides for prevention and treatment of disease and parasites. Our results suggest that seasonal co-application of these medicines to bee hives could increase the adverse effects of these and perhaps other pesticides. Our results also demonstrate the utility of a mechanism-based strategy. By identifying pesticides and apicultural medicines that are substrates and inhibitors of xenobiotic transporters we prioritize the testing of those chemical combinations most likely to result in adverse interactions.

## Introduction

Honey bees are in trouble. Widespread depopulation of colonies often characterized by high overwintering losses has occurred since at least 2006 in the United States, threatening the sustainability of North American apiculture. Despite considerable effort, no single cause of the phenomenon called colony collapse disorder (CCD) has been identified, though associations of several pathogens and parasites appear to increase the risk of colony collapse [1], [2]. Pesticides are also among the suspected contributing factors of colony collapse both because bees encounter a diverse array of pesticides when foraging and because more than 120 different pesticides have been found within bee hives [2], [3], [4], [5], [6]. Some pesticides have received extra scrutiny, notably the acaricides coumaphos and τ-fluvalinate, applied to bee hives for control of parasitic varroa mites, and the widely used neonicotinoid insecticides. These acaricides are applied directly to bee hives, accumulate in wax and were found in nearly all hives recently tested in both N. American and France [5], [6]. The neonicotinoids (especially imidacloprid) are of concern because they are toxic to honey bees, used on many crops and ornamental plants, and they tend to be systemically distributed within treated plants, potentially contaminating nectar and pollen of treated and rotational crops not initially treated with these products [7], [8], [9], [10].

Although pesticide drift and overdosing cause accidental bee kills no single pesticide has been directly implicated with widespread overwintering losses or CCD [2], [5]. It remains possible however, that combinations of toxins may cause adverse additive or synergistic effects that would be difficult to detect through surveys of beekeepers or analysis of their apiaries without dedicated multifactorial analysis. It has been shown, for example, that the toxicity to bees of some pyrethroid and neonicotinoid insecticides increases significantly when combined with certain fungicides [11], [12]. Similarly, Johnson et al. [13] found that coumaphos and τ-fluvalinate each synergize the other's toxicity to honey bees, perhaps through competitive inhibition of the metabolic enzymes that detoxify those pesticides. Given the many pesticides that bees encounter there may be adverse combinations of them eroding hive health in both subtle and dramatic ways.

The problem, of course, is the large number of potentially adverse pesticide combinations which prevents evaluation of all, or even most, combinations of them. This problem challenges our ability to anticipate the risks associated with bee's exposure to a novel pesticide or to identify combinations of toxins contributing to a colony collapse. If we could identify mechanisms of the honey bee xenobiotic metabolism and excretion systems that systematically mediate multiple-toxin interactions, we could reduce the overwhelming number of candidate pesticide interactions to a smaller set of compounds that are substrates or inhibitors of the most predictive mechanisms.

The membrane-bound transporter proteins from the ABC transporter family of proteins are found in all phyla [14], [15]. The xenobiotic transporters in this family actively shuttle toxins across cell membranes to reduce the intracellular toxin and metabolite concentrations. Working in concert with metabolic enzymes, these transporters mediate a baseline tolerance to a diverse array of toxins including numerous drugs, pesticides and phytochemicals [16], [17]. Several of these transporters, especially members of the ABCB, ABCC, and ABCG subfamilies of transporters (referred to here as Multiple Drug Resistance, or MDR transporters), are of medical importance, playing a role in resistance to multiple cancer and anti-parasite drugs [17], [18], [19].

MDR transporters are relatively unstudied in insects, and completely neglected in honey bee toxicology. These transporters act in several insect tissues, including the cuticle [20], malpighian tubules [21], [22], midgut [23] and at the blood-brain barrier [24], [25] to transport toxins, including pesticides, towards excretion [17]. The honey bee genome contains genes coding for orthologues of these proteins, which presumably protect bees from toxins as they do in *Drosophila melanogaster* [24], [26], [27], chironomid flies [28], mosquitoes [29], *Heliothis virescens* (tobacco budworm) [20] and *Manduca sexta* (tomato hornworm) [21], [25]. It seems reasonable therefore to consider the role that these proteins play in honey bee tolerance of pesticides and to begin an analysis of potentially inhibitory compounds that bees commonly encounter.

The most well studied MDR transporter, p-glycoprotein (p-gp), has both a diverse range of substrates and is inhibited by an array of drugs, pesticides and plant compounds [17]. This inhibition is a mechanism by which MDR transporters would cause adverse interactions among many chemicals; one compound inhibits the transporters thereby increasing sensitivity to other toxic substrates. The drug verapamil is a potent inhibitor of p-gp and possibly other MDR transporters [30], [31]. It is frequently used as the standard inhibitor of p-gp where it increases the sensitivity of treated cells, tissues or organisms to toxic transporter substrates [17], [18], [26]. Here we use verapamil inhibition to determine if 5 pesticides are substrates of MDR transporters and therefore potentially synergized by other inhibitors more likely to be encountered by honey bees. Remarkably, three widely used in-hive pesticides and medications (the previously mentioned acaricides coumaphos and τ-fluvalinate and the antibiotic oxytetracycline) are known substrates and/or inhibitors of mammalian p-gp [31], [32], [33]. We suspect that these in-hive medications and pesticides may be interacting with bee's MDR transporters, increasing their sensitivity to these and perhaps other pesticides and toxins. The frequent contamination of hive wax with these acaricides [6] and routine treatment of hives with oxytetracycline [34], [35], [36], [37] undoubtedly increases the exposure of bees to these compounds, with potentially significant consequences if they are indeed substrates or inhibitors of honey bee MDR transporters.

Interaction of neonicotinoid insecticides with insect MDR transporters has not yet been reported. Because of the likelihood of exposure of bees to these insecticides we ask if the neonicotinoid insecticides imidacloprid, acetamiprid and thiacloprid are substrates of honey bee MDR transporters. Evidence of neonicotinoid processing by MDR transporters would be significant because inhibition of those transporters could cause mortality at lower doses than normally expected for individual compounds.

## Results

**Figure 1:**
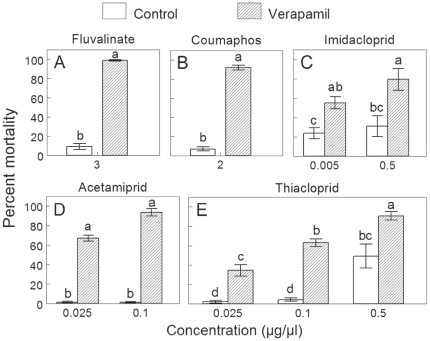
Verapamil synergizes honey bee mortality by five acaricides/insecticides. Mean mortality (±SE) of honey bees (average of 24 and 48 h) following topical (A, B) and oral (C, D, E) exposure to pesticides. Bees were pre-fed sucrose or sucrose+verapamil (1 mM) solution. For each pesticide, different letters indicate significant differences between treatments (*p*<0.05).

When fed to bees verapamil significantly increased the toxicity of all 5 acaricides/insecticides. Mean mortality of young worker bees topically treated with the acaricides coumaphos or τ-fluvalinate was significantly higher when bees were pretreated with verapamil (Fig. 1, Table 1). Control mortality following topical application of acetone was 0% for both sucrose and sucrose+verapamil fed bees. Acute oral toxicity was also significantly higher for all three neonicotinoids (acetamiprid, thiacloprid, imidacloprid) when bees were pretreated with verapamil (Fig. 1, Table 2). Increased mortality at higher concentrations and at the later end point (48 h) was observed for thiacloprid, and at 48 h for imidacloprid. The effect of verapamil pretreatment did not differ among concentrations of these insecticides (Table 2). Control mortality of sucrose only and sucrose+verapamil cohorts averaged 2–3%.

**Table 1:** Repeated-measures analysis of variance of honey bee mortality.

	Pesticide treatment (Pretreatment)								
	Coumaphos (Verapamil)			τ-Fluvalinate (Verapamil)			Coumaphos (OTC)		
	df	F	*p*	df	F	*p*	df	F	*p*
Pretreatment	1,14.5	61.89	<0.0001	1,10	57.77	<0.0001	1,11	10.83	0.0072
Time	1,11.2	3.91	0.07	1,10	1.46	0.26	1,9.8	10.64	0.0088
Pretreatment×Time	1,11.2	3.20	0.10	1,10	1.46	0.26	1,9.8	1.66	0.2277

Bees were pretreated with verapamil, oxytetracycline (OTC), or sucrose syrup then treated with the acaricides coumaphos or τ-fluvalinate.

**Table 2:** Repeated-measures analysis of variance of honey bee mortality.

	Imidacloprid			Acetamiprid			Thiacloprid		
	df	F	*p*	df	F	*p*	df	F	*p*
Pretreatment	1,28	17.78	0.0002	1,12	128.54	<0.0001	1,24	65.53	<0.0001
Concentration	1,28	2.75	0.11	1,12	0.26	0.62	1,24	27.93	<0.0001
Time	1,28	43.12	<0.0001	1,12	1.24	0.29	1,24	94.97	<0.0001
Pretreatment×Concentration	1,28	0.80	0.38	1,12	0.27	0.61	1,24	2.39	0.11
Pretreatment×Time	1,28	1.72	0.2	1,12	0.63	0.44	1,24	53.31	<0.0001
Concentration×Time	1,28	0.66	0.42	1,12	1.02	0.33	1,24	58.17	<0.0001
Pre×Conce×Time	1,28	3.51	0.07	1,12	0.80	0.39	1,24	69.75	<0.0001

Bees were pretreated with verapamil or sucrose syrup and then fed one of three neonicotinoid insecticides.

**Figure 2:**
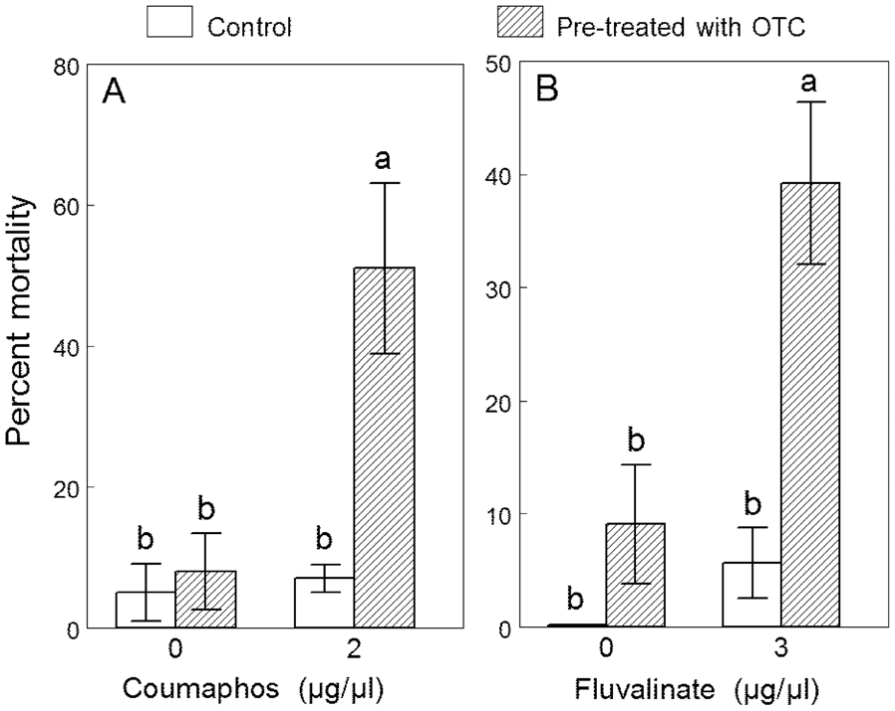
Oxytetracycline (OTC) synergizes honey bee mortality by in-hive acaricides. Mean mortality (±SE) of honey bees pre-fed sucrose solution (50%) or sucrose+oxytetracycline (1.4 mM) and topical application of (A) coumaphos (average of 24 and 48 h) and (B) τ-fluvalinate (24 h). For each pesticide, different letters indicate significant differences between treatments (*p*<0.05).

Oxytetracycline significantly increased the mortality of bees exposed to coumaphos and τ-fluvalinate (Fig. 2). For comparison with the verapamil synergism reported above, mean mortality of bees treated with 2 ug/ul coumaphos increased from 7% (n = 4 cages) to 51% (n = 4 cages) following feeding of OTC (1.4 mM), a significant but smaller increase than that caused by verapamil (Fig. 2A,Table 1). OTC feeding increased the mortality of bees treated with 3 ug/ul τ-fluvalinate from 5.6% (n = 10 cages) to 39% (n = 8 cages) (Fig. 2B, *p* = 0.002). Mean mortality of cohorts fed OTC alone were below 10% and were not significantly different from those fed sucrose alone (Fig. 2).

## Discussion

Here we provide the first evidence that the MDR transporter(s) inhibited by verapamil play a role in protecting honey bees from pesticides, and that the acaricides coumaphos and τ-fluvalinate, and 3 neonicotinoid insecticides are substrates of these transporters in insects. The observation that coumaphos and τ-fluvalinate are substrates of honey bee p-gp or another MDR transporter was anticipated from previous study of mouse cells, and suggests that insect and mammalian MDR transporters share substrates. Clearly, the abundance of these pesticides found in the wax and pollen of bee hives [6] coupled with evidence that their toxicity to bees is increased through inhibition of MDR transporters implicates them as toxins of interest in any multifactorial explanation of high overwintering colony losses.

This is the first report that neonicotinoid insecticides are substrates of insect MDR transporters. In efforts to protect honey bees, energetic opposition to the neonicotinoids has arisen in North America and Europe, but direct implication of them in overwintering losses has not been sustained by recent research [2], [6]. Estimates of the environmental exposure of bees to imidacloprid are typically low relative to the LD_50_ [6], and studies have not demonstrated hive-level consequences of imidacloprid contamination [38]. Our results suggest that inhibition of MDR transporters may reduce the LD_50_ of neonicotinoids possibly amplifying acute and chronic effects to bees at lower concentrations.

The large increases in sensitivity to pesticides by inhibition of MDR transporters and the chemical diversity of the synergized pesticides suggest that these transporters may mediate adverse synergisms of diverse toxins in bees. Because of its clinical importance in human health, proven and candidate p-gp substrates and inhibitors of many types have been identified [16], [17]. Knowledge of these compounds may help us identify chemicals likely to interact with honey bee MDR transporters. In the first application of this mechanism-based strategy to honey bees, we uncover a significant negative interaction among three medications routinely applied to bee hives [35], [36], [37]. OTC, coumaphos and τ-fluvalinate are all known to interact with mammalian p-gp [31], [32], [33]. If honey bee transporters behave similarly, we would expect increased toxicity following co-application of a toxic transporter substrate and an inhibitor. As anticipated, concentrations of OTC similar to those applied to bee hives increased bee's sensitivity to both coumaphos and τ-fluvalinate. OTC is applied to bee hives in the late fall and/or early spring, often in tandem with one of the acaricides [36]. Our results suggest that co-application of these compounds could increase the likelihood of intoxication by the acaricides and other pesticides contaminating beeswax and food stores. These results raise the possibility that adverse interactions of medications (such as OTC) and pesticides (coumaphos and τ-fluvalinate) contribute to the loss of honey bee colonies during the winter or early spring, a common feature of CCD. Although the per-bee concentration of OTC used here is similar to field application rates, the pesticide concentrations are higher than those found in bee hives (see [6]). Therefore, although we have demonstrated that verapamil and OTC increase bee's sensitivity to these pesticides in acute laboratory bioassays, additional testing of lower pesticide concentrations over longer time periods is necessary to fully understand the field relevance of these interactions. Additional work is also required to directly demonstrate that OTC inhibits p-gp or other efflux transporters in honey bees. Nevertheless, we show here using OTC and the acaricides as an example, how identification of MDR transporter substrates and inhibitors can highlight potentially dangerous chemical combinations and improve the assessment and management of toxicological risks faced by honey bees.

## Materials and Methods

### Insects

Bees were collected for laboratory bioassays from newly established colonies reared on new frames and freshly drawn comb. Colonies were not treated with apicultural medications or pesticides. Frames with emerging workers were taken from hives and placed into dark growth chambers maintained at 33±2°C and (70–80%) RH. Newly-emerged bees were collected from the frames daily and maintained in groups of 20–30 in 80×100 mm metal mesh cages capped at each end by standard polystyrene petri dishes. Bees were fed sucrose solution (50%; w∶v) through 1 mm holes from a 2.0 ml microfuge tube.

### Chemicals

Terramycin (oxytetracycline, 5.5% soluble powder, Pfizer) was purchased from Dadant and Sons (Hamilton, Illinois). Coumaphos, τ-fluvalinate (both technical grade) and verapamil were purchased from Sigma-Aldrich Inc. (St. Louis, MO). Commercial formulations of imidacloprid (Admire Pro) and thiacloprid (Calypso) were provided by Bayer CropScience (Durham, NC), and acetamiprid (Assail) was provided by United Phosphorous Inc. (King of Prussia, PA).

### Drug pretreatments

Verapamil (1 mM) and oxytetracycline (OTC, 1.4 mM) were incorporated into 50% sucrose solutions for oral dosing of 1–3 day old workers. Preliminary feeding trials of 1 mM solutions of oral verapamil revealed no toxicity. The 1.4 mM concentration of OTC provides a per-bee exposure comparable to that of the label-recommended dosage of 600 mg applied to a hive containing 12,000 bees—a typical colony size entering winter [39]. Sucrose-drug solutions were made fresh every 3 days and the vials supplying each cage were replaced as needed.

### Topical bioassays of insecticides/acaricides

Cohorts of 3–6 day old workers pretreated by feeding with the two sucrose-drug solutions were anesthetized with CO_2_, and 1 ul of coumaphos (2 ug/ul)or τ-fluvalinate (3 ug/ul) in acetone (or acetone alone for controls) was applied to the dorsal thorax of each bee using an ISCO microapplicator driving a 1/4 cc tuberculin syringe. After application, bees were returned to cages containing the sucrose-drug or sucrose-only solution. Mortality of bees in each cage was recorded at 24 and 48 h. 5–10 replicate cohorts of 25 bees were tested for each acaricide - pretreatment combination.

### Oral bioassays of insecticides

Pre-fed cohorts of 3–6 day old workers were fed sucrose syrup containing one of the neonicotinoids. Mortality of each cage was recorded at 24 and 48 hours. Imidacloprid was tested at 5 and 50 ng/ul, acetamiprid at 25 and 100 ng/ul, and thiacloprid at 25, 100 and 500 ng/ul. These concentrations caused low-intermediate mortality of bees fed sucrose-only solution in preliminary range-finding experiments. 2–13 replicate cohorts of 25 bees were tested for each toxin concentration - pretreatment combination.

#### Analysis

The effects of verapamil or OTC pretreatment on insecticide/acaricide mortality were tested using a repeated measures analysis of variance (Proc Mixed, SAS). Following transformation (arcsine square-root), mortality was analyzed with a model that included pretreatment, insecticide concentration if multiple levels were used, and time endpoints (24 and 48 h) as fixed factors to assess the main effects and their interactions. Because only mortality at 24 h was available, analysis of τ-fluvalinate combined with OTC, was performed using a simple t-test, comparing the τ-fluvalinate and the τ-fluvalinate+OTC treatments.
